# Budesonide-Loaded Pectin/Polyacrylamide Hydrogel for Sustained Delivery: Fabrication, Characterization and In Vitro Release Kinetics

**DOI:** 10.3390/molecules26092704

**Published:** 2021-05-05

**Authors:** Manisha Pandey, Hira Choudhury, Sahleni Kaur D/O Segar Singh, Naveenya Chetty Annan, Subrat Kumar Bhattamisra, Bapi Gorain, Mohd Cairul Iqbal Mohd Amin

**Affiliations:** 1 Department of Pharmaceutical Technology, School of Pharmacy, International Medical University, Bukit Jalil, Kuala Lumpur 57000, Malaysia; 2 Centre for Bioactive Molecules and Drug Delivery, Institute for Research, Development and Innovation, International Medical University, Kuala Lumpur 57000, Malaysia; 3 Bachelor of Pharmacy Student, School of Pharmacy, International Medical University, Kuala Lumpur 57000, Malaysia; sahlenikaur95@gmail.com (S.K.D.S.S.); naveenya_vee@yahoo.com (N.C.A.); 4 Department of Life sciences, School of Pharmacy, International Medical University-Bukit Jalil, Kuala Lumpur 57000, Malaysia; subratkumar@imu.edu.my; 5 School of Pharmacy, Faculty of Health and Medical Sciences, Taylor’s University, Subang Jaya 47500, Malaysia; bapi.gn@gmail.com; 6 Center for Drug Delivery and Molecular Pharmacology, Faculty of Health and Medical Sciences, Taylor’s University, Subang Jaya 47500, Malaysia; 7 Centre for Drug Delivery Technology, Faculty of Pharmacy, Universiti Kebangsaan Malaysia, Jalan Raja Muda Abdul Aziz, Kuala Lumpur 50300, Malaysia; mciamin@ukm.edu.my

**Keywords:** ulcerative colitis, hydrogel, pectin, colon drug delivery, pH-sensitive

## Abstract

A single ulcerative colitis (UC) is a chronic inflammatory bowel disease (IBD) that causes inflammation of the colonic mucosa at the distal colon and rectum. The mainstay therapy involves anti-inflammatory immunosuppression based on the disease location and severity. The disadvantages of using systemic corticosteroids for UC treatment is the amplified risk of malignancies and infections. Therefore, topical treatments are safer as they have fewer systemic side effects due to less systemic exposure. In this context, pH sensitive and enzymatically triggered hydrogel of pectin (PC) and polyacrylamide (PAM) has been developed to facilitate colon-targeted delivery of budesonide (BUD) for the treatment of UC. The hydrogels were characterized by Fourier transform infrared spectroscopy (FTIR), X-ray diffraction (XRD), swelling ratio, and drug release. FT-IR spectroscopy confirmed the grafting as well loading of BUD in hydrogel. XRD showed the amorphous nature of hydrogel and increment in crystallinity after drug loading. On the other hand, SEM showed that the hydrogels exhibited a highly porous morphology, which is suitable for drug loading and also demonstrated a pH-responsive swelling behaviour, with decreased swelling in acidic media. The in-vitro release of BUD from the hydrogel exhibited a sustained release behaviour with non-ficken diffusion mechanism. The model that fitted best for BUD released was the Higuchi kinetic model. It was concluded that enzyme/pH dual-sensitive hydrogels are an effective colon-targeted delivery system for UC.

## 1. Introduction

Ulcerative colitis (UC) is a chronic inflammatory bowel disease (IBD) that causes inflammation of the colonic mucosa at the distal colon and rectum. Symptoms include bloody diarrhea, abdominal pain, tenesmus, and unexplained weight loss [1]. The mainstay therapy involves anti-inflammatory immunosuppression based on the disease location and severity. UC treatments comprise 5-aminosalicylic acid (5-ASA) drugs, corticosteroids, and immunosuppressants [2].

For a long time, the treatment options for mild to moderate UC were constricted to oral and rectal 5-ASA formulations as well as rectal corticosteroid preparations [3]. The disadvantage of using systemic corticosteroids for UC treatment is the amplified risk of malignancies and infections [4,5]. Topical treatments are safer as they have fewer systemic side effects due to lesser systemic exposure [5].

An alternative to prednisolone is a local corticosteroid, budesonide. Budesonide is related structurally to 16α-hydroxyprednisolone and has anti-inflammatory properties [6]. Budesonide has a 15 times higher affinity for glucocorticoid receptors and better topical potency that is almost five times more than that of prednisone [7]. The drug is cleared rapidly through high first-pass metabolism in the liver, resulting in low systemic bioavailability of only about 10% [6,7]. Due to this limited systemic exposure of budesonide, its systemic side effects are much lower than conventional corticosteroids. When compared against prednisolone and other corticosteroids at clinically equivalent doses, budesonide shows hypothalamic-pituitary-adrenal axis suppression and steroid-associated side effects that are significantly lower [6,8]. Studies show that the limitation of its low bioavailability can be overcome by developing a colon-targeted delivery system for budesonide [9].

In 2013, the oral extended-release formulation and rectal application of budesonide gained approval by the US Food and Drug Administration (FDA) for remission induction in patients with mild to moderate UC [10]. At present, there are three budesonide formulations, two of which are standard capsules both designed to release the drug only at the distal ileum and proximal colon, which are not UC target sites [11]. The latest commercially available formulation, the budesonide multimatrix (MMX) capsule, was designed in the year 2014 to release the drug throughout the entire colon [7,12]. This formulation has been shown to have significant remission induction in patients with UC [13].

Budenofalk^®^ and Entocort^®^ are examples of a strategy developed by coating budesonide with a pH-sensitive polymer [13,14].

Similarly, polysaccharide-based delivery systems have been extensively studied and proven to be a promising colon-targeted delivery system [15,16,17]. Polysaccharides are abundantly available and can be easily modified both chemically and biochemically [16,18]. A unique characteristic of these polysaccharides is the ability to remain as a whole in the acidic nature of the stomach as well as in the environment of the small intestines. Conversely, polysaccharides are degraded by microflora that is found in the colon making it a desirable carrier system for polypeptide drugs used to treat colonic diseases, such as UC [19].

Ye Liu and Hong Zhou conducted a study in which a natural polysaccharide, guar gum was used to develop a budesonide colon-targeted system. This study investigated the in-vitro drug release in simulated gastric fluid (pH 1.2) and simulated intestinal fluid (pH 7.5). It revealed a slower, sustained release pattern of budesonide. This shows that budesonide-loaded natural polysaccharide formulation was able to withstand the acidic nature of the stomach and intestinal environment due to the gel-forming ability of polysaccharides. A similar study was conducted by Krishnaiah et al. whereby a formulation of compression coated guar gum tablets was developed [20].

Since the colon is the target site for UC treatment, a colon-targeted hydrogel will be a good novel drug-delivery system for budesonide. Hydrogels are three-dimensional hydrophilic polymeric networks synthesized by physical and chemical crosslinking methods [21,22].

In this project, pH-sensitive enzyme-triggered hydrogels are synthesized using pectin and PAM to facilitate colon-targeted delivery of budesonide for the treatment of UC. The polysaccharide, pectin, helps in colon targeting as it remains intact in the upper gastrointestinal tract and only degrades at the colon by colonic enzymes [23,24] while PAM acts as a pH-sensitive polymer which is insoluble at lower pH levels but becomes increasingly soluble as the pH increases [25]. The optimization of pH-sensitive pectin/PAM hydrogel was carried out based on gel fraction, swelling behaviour, drug loading, and release.

## 2. Materials and Methods

### 2.1. Materials

Acrylamide (AM), *N*,*N*′-methylene bisacrylamide (MBA), Potassium persulfate (KPS), and Pectin (PC) were purchased from Sigma-Aldrich. Sodium hydroxide, hydrochloric acid, and other reagents used in this study were of analytical grade and used without further purification.

### 2.2. Methods

#### 2.2.1. Preparation of Pectin/Polyacrylamide Hydrogel

The hydrogels were prepared using acrylamide (AM) and pectin (PC) with the aid of an initiator and a crosslinker. Different amounts of AM were dissolved in 20 mL of PC solution followed by the addition of potassium persulfate (initiator) and MBA (crosslinker). The resultant mixture was placed in the oven at 60 °C to allow the formation of hydrogel. The prepared hydrogels were immersed in distilled water for 120 h to remove any excess reactants. The hydrogels were dried in the oven at 60 °C for 24 h.

#### 2.2.2. Determination of Gel Fraction

The freshly prepared hydrogels were cut into discs using a mould of 1.0 cm diameter. The discs were placed in the oven at 60 °C to obtain a constant weight (*G*0). The dried discs were soaked in distilled water at room temperature for 7 days to extract any remaining AM or PC. The extracted discs were re-dried in an oven at 60 °C to obtain a constant weight (*G*1). The percentage of gel fraction (% *GF*) was calculated using the equation below:

(1) % GF=(G1|G0)×100

#### 2.2.3. FT-IR Spectroscopic Analysis

The IR spectra of samples were recorded using Perkin Elmer FT-IR Spectra 2000 at room temperature. KBr-disk method was used to prepare the samples. The samples were investigated over the range of 4000–400 cm^−1^.

#### 2.2.4. X-ray Diffraction

X-ray diffractogram of the hydrogel was characterized by X-ray diffractometer (DB-Advance, Bruker) using CuKά radiation at 40 kV and 50 mA in differential angle range of 5–70° 2θ.

#### 2.2.5. Differential Scanning Calorimetry (DSC)

DSC thermogram of hydrogel was recorded using differential scanning calorimeter (Melter Toledo DSC Stare). About 3–4 mg of each sample was placed in the pan and heated over the temperature range of 20–200 °C with a heating rate of 10 °C/min.

#### 2.2.6. Morphological Analysis

Morphology of hydrogels were studied at different pH. In this regard, dried hydrogel disk (weight approx. 100 mg) were soaked in various phosphate buffer pHs (1.5, 5.8, and 7.4) for 24 h. Afterward hydrogel disks were washed with water and freeze-dried for morphology analysis. The external morphology and porous structure of the hydrogels were analysed using scanning electron microscopy (SEM) (SC500; BioRad, London, UK). The samples were mounted on an aluminum stub and coated with gold in a sputter coater under an argon atmosphere.

#### 2.2.7. Swelling Studies

The swelling behaviour of the hydrogels was studied under various phosphate buffer pHs (1.5, 5.8, and 7.4). The dried extracted hydrogel disk (*Gd*) (approx. weight 100 mg) was soaked in 50 mL of media. At fixed time intervals (30 min, 1 h, 2 h, 4 h, 6 h, 8 h, and 24 h), the weight of the swollen hydrogel was recorded (*Gs*). The study was conducted in triplicates. The percentage swelling (% *SR*) was calculated using the equation below:

(2) % SR=(Gs−Gd|Gd)×100

#### 2.2.8. Drug Loading

The dried hydrogels disk (weight 100 mg) was loaded with budesonide by placing the hydrogel disk (approx. 100 mg) in 5 mL of drug solution (10 mg) in alcohol for 8 h. The swollen hydrogels were removed and dried to a constant weight. The absorbance of the remaining solution was measured using a spectrophotometer and the concentration of the drug remaining was determined. Drug loading (*DL*) and drug entrapment efficiency (*EE*) were calculated using the equations below:

(3) EE=(Wo−Wf|Wo)×100

(4) DL=(Wdg|Wg)×100.

where *W*_0_ is total amount of drug in the solution before loading, *Wf* is total amount of drug in the solution after loading, *Wdg* is the amount of drug incorporated into the hydrogel, and *Wg* is the weight of the dried hydrogel.

#### 2.2.9. In-Vitro Drug Release Study

The drug-loaded hydrogels disk (approx. weight 108 mg) was immersed in 50 mL hydrochloric acid buffer pH 1.5 for 2 h. Then, the hydrogels were immersed in phosphate buffer pH 7.4 for 48 h. The drug-loaded hydrogels were also immersed in buffer pH 7.4 alone to compare drug release profiles between the two systems. The dissolution media was kept at 37 °C with constant agitation (50 rpm). At fixed time intervals, aliquots were withdrawn and substituted with fresh media. The concentration of the drug was measured at 272 nm using the spectrophotometer method. The results are demonstrated as percentage release over time.

## 3. Results and Discussion

### 3.1. Synthesis of Hydrogels

**Figure 1:**
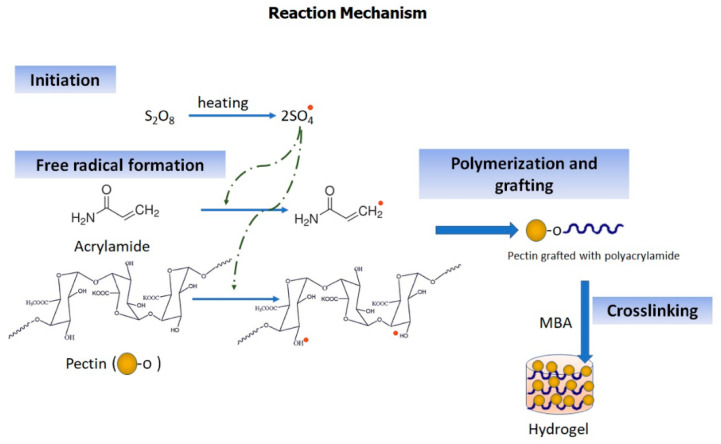
Reaction mechanism of hydrogel.

The pH-sensitive PC/PAM hydrogel was prepared by grafting AM on PC with the aid of the initiator potassium persulfate followed by cross-linking with MBA. The reaction begins with the generation of sulfate ion radicals (SO^4−^) from the initiator, potassium persulfate, upon conventional heating at 60 °C. In the presence of heat, these radicals react with water molecules to produce hydroxyl free radicals which were then used for the initiation of the polymerization process. The hydroxyl free radicals act on PC and AM and generate active sides on both the reactants. As a result, the reactants bind to form long chains of grafted polymers. Finally, to crosslink the polymer chains formed, MBA was added (Figure 1). Appearance of hydrogel was visual by Figure S1.

#### 3.1.1. Optimization of Pectin/Acrylamide Ratio

To optimize the PC to AM ratio, various concentrations of PC and AM were added into the formulations while keeping the ratio of MBA to KPS constant (Table 1) and the ratio was optimized on the basis of gel fraction and percentage swelling. The hydrogel gel fraction (GF) represents the degree of cross-linking of the grafted polymer. Cross-linking ensures that the hydrogel does not dissolve and only expands in aqueous media. A greater degree of cross-linking results in higher GF. The results of the corresponding gel fraction of hydrogels (%) are shown in Table 1.

As observed from Table 1, GF increased with an increase in the amount of AM (1.5−2.8 g) which contribute to higher grafting and polymerization at higher concentrations of AM. Yin et al. reported similar findings where poly(acrylic acid-co acrylamide)/carboxymethyl chitosan hydrogels were developed, and gel fraction was found leading to poly(acrylic acid-co acrylamide) in the reaction mixture [26]. Conversely, when the amount of PC was increased from 0.5 g to 1 g, formulations of gel fraction deceased, as evident with 0.5 g PC yielding higher GF, as demonstrated in formulations 1A (58.90%), F3 (77.23%), and 1D (81.08%) compared to formulation contains 1 g PC. GF of the hydrogel is dependent on both AM and PC concentrations. The highest GF was recorded with 1 D formulation. However, this was not statistically different from F3 GF.

**Figure 2:**
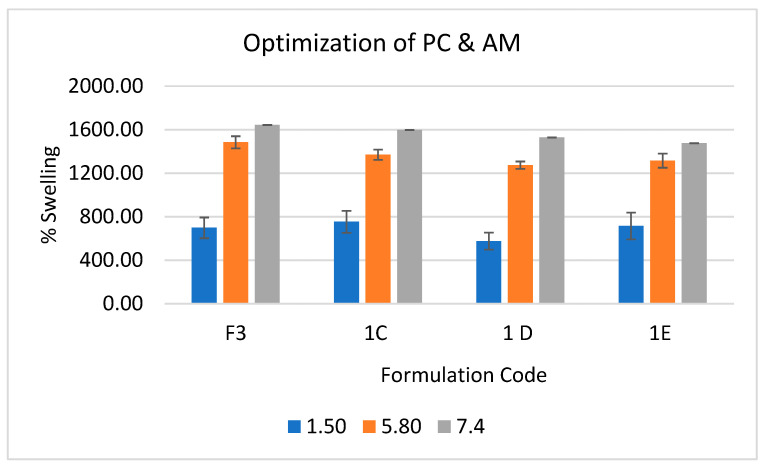
Swelling behaviour of hydrogels containing different PC/AM ratio at various phosphate buffer pH (1.5, 5.8, 7.4).

Secondly, swelling ratio (SR) was estimated for optimization of hydrogels which represent its water holding capacity. It is the key factor for drug loading and release. The super-absorbency of the PC/PAM hydrogel is due to ionic carboxylate and non-ionic carboxamide functional groups. The presence of ionic groups in polymer chains increase SR because these ions are more toughly solvated rather than non-ionic groups in the aqueous media. The pH-sensitivity of hydrogels was studied in phosphate buffer solutions (pH 5.8 and 7.4) and also using hydrochloric acid (pH 1.5). As seen in Figure 2, the swelling behaviour of hydrogels indicated pH-dependent swelling. The swelling percentage increased from pH 1.5 to 7.4 with maximum swelling at pH 7.4. However, there was no drastic increment from pH 5.8 to 7.4. This pattern could be due to the presence of carboxylate ions in the PAM chain. At pH < 4.7, the carboxylate group is protonated, whereas at pH 5–7, deprotonation happens, creating an electrostatic repulsive force and instigating greater swelling. Additionally, the increment of AM concentration in hydrogels leads to a lower percentage of swelling, this may contribute to higher polymerization and crosslinking density. From the GF and SR results, formulation 3 (F3) was determined to be the optimised formulation.

#### 3.1.2. Optimization of MBA/KPS Ratio

To optimize the MBA to potassium persulfate ratio based on GF and SR, various concentrations of MBA and KPS were added into the formulations while keeping the ratio of AM to PC constant. The results of the corresponding gel fraction of hydrogel (%) are shown in Table 2. It was observed that by increasing the concentration of MBA (F1–F5), the % gel fraction increased from 60.23% to 79.47%. This suggests that the greater the amount of crosslinker added to the initial solution, the more cross-linked hydrogels will be formed, and hence a higher gel fraction of hydrogel. Similarly, the % gel fraction of hydrogel was found to be greater with increasing concentration of potassium persulfate. For instance, it can be seen that formulation 7 with the highest amount of potassium persulfate has a gel fraction of 79.13% which is significantly different from formulation 6 (63.4%) having the lowest amount of KPS. However, an insignificant difference was found when the concentration increased from 20 mg to 50 mg in 20 mL of reaction mixture.

**Figure 3:**
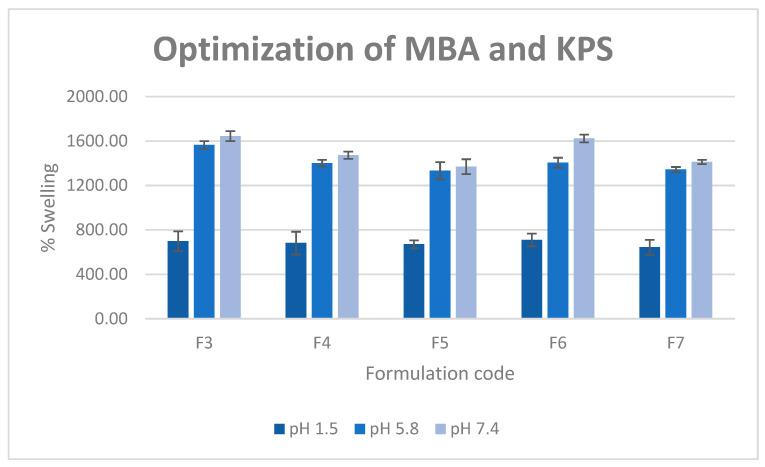
Swelling behaviour of hydrogels contains different amount of MBA and KPS at various phosphate buffer pH (1.5, 5.8, 7.4).

It is assumed that when the initiator concentration is increased, more radicals are produced from the initiator and utilised to produce growing monomer radicals. This eventually results in the maximum gel fraction of hydrogel, and at some point, saturation occurred that led to an insignificant increment in GF. Additionally, Figure 3 showed a lower SR value with increment in MBA and KPS concentration due to high generation of free radical and high crosslinking which restrict the swelling of the hydrogel. The % gel fraction and SR data pointed towards formulation 3 as the optimized formulation due to the high gel fraction and small statistical difference.

### 3.2. Characterization of Budesonide Loaded Optimized Hydrogel

After optimization of polymer, crosslinker, and initiator, optimized formulation (F3) was loaded with budesonide and characterized on the different parameters to evaluate compatibility, surface morphology, loading and release.

#### 3.2.1. FT-IR Spectroscopic Analysis

**Figure 4:**
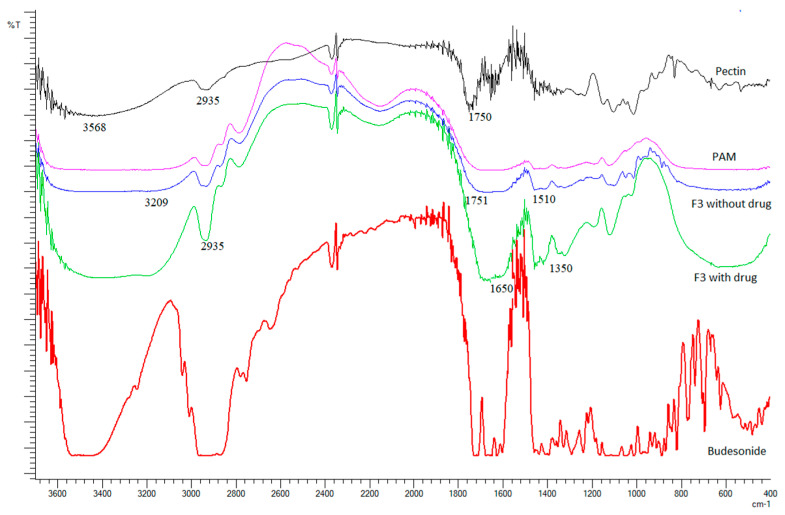
FTIR spectra of pure PC, polyacrylamide, F3 without drug, F3 with drug and budesonide.

Figure 4 shows the FTIR spectra of pure PC, PAM, F3 without budesonide, F3 with budesonide and budesonide. The spectrum of pure PC showed an absorption peak at 3568 cm^−1^ indicating the stretching of –OH groups. The peak observed at 2935 cm^−1^ is attributed to –C–H stretching vibration. The peak at 1750 cm^−1^ represents > C=O stretching vibrations on account of the presence of –COOCH3 group. The peaks at 1458 cm^−1^ and 1419 cm^−1^ could be due to –CH2 vibration and –OH bending vibration, respectively. The presence of a –CH–OH group in aliphatic cyclic secondary alcohol was shown by the peak at 1149 cm^−1^. Next, according to the spectra of hydrogel with and without drug, common peaks between 3209 and 3250 cm^−1^ were observed in hydrogels. These peaks suggested –OH and –NH stretching. It was also observed that these peaks are more intense in the spectra of F3 with the drug compared to the spectra of F3 without the drug. This is due to the presence of –OH groups in the structure of budesonide which indicates that the hydrogel has been loaded with the drug successfully. Next, the peak at 1650 cm^−1^, as seen in both hydrogel spectra, indicated amide-I band (C=O stretching vibration) of the amide group which is the characteristic group of PAM. Similarly, amide-II bands within the range of 1510–1533 cm^−1^ were due to C–N stretching vibrations as well as N–H bending. The presence of these amide groups in the spectra of the hydrogels indicates the formation of grafted polymer. The peak at 1751 cm^−1^ could be assigned to free carboxylic groups while the peak at 2935 cm^−1^ corresponds to C–H stretching of–CH2 groups. Lastly, the peaks of –CH bending of –CH2 group lie at 1350–1460 cm^−1^.

#### 3.2.2. X-ray Diffraction

**Figure 5:**
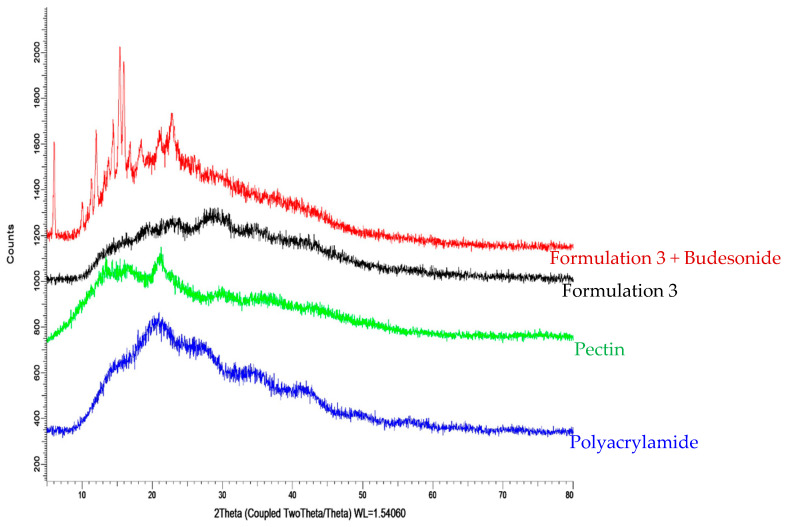
X-RD analysis of pectin, polyacrylamide (PAM), loaded hydrogel (Formulation 3 + drug) and unloaded hydrogel (Formulation3).

From Figure 5, the X-ray diffractogram of pure PC shows two broad peaks at 2θ equals to 15.10°θ and 21.36°θ, indicating the amorphous nature of PC. Broad peaks can also be observed from the X-ray diffractogram of PAM at 2θ equals to 16.02°θ, 21.50°θ, 27.42°θ, 34.37°θ, 41.13°θ, and 52.87°θ, indicating the amorphous nature of PAM. As a result of both parent compounds being amorphous, the resultant grafted polymer (hydrogel) had an increased amorphous nature.

**Figure 6:**
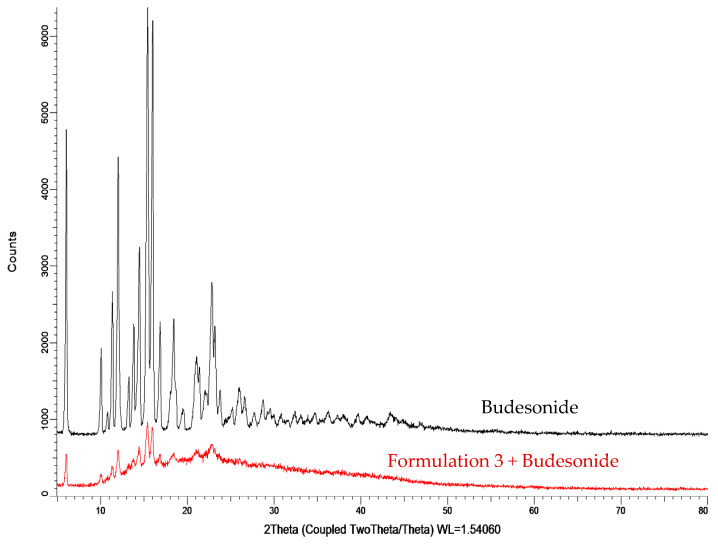
X-RD analysis budesonide and the loaded hydrogel.

The X-ray diffractograms of the unloaded hydrogels show broad peaks between 15oθ–40oθ, which can be attributed to the interactions between amorphous PC and PAM. Diffractogram of pure AM shows sharp crystalline peaks at 2θ equals to 12.02oθ, 19.58oθ, 24.13oθ, 28.69oθ, and 36.48oθ, which clearly indicate the crystalline behaviour of AM. As the hydrogels were highly amorphous and devoid of any sharp crystalline peaks, this indicates that AM was fully polymerized and properly extracted from the grafted polymer. Moreover, Figure 6 indicates crystalline nature of budesonide with many intense peaks in X-ray diffractogram between 10oθ to 25oθ. There is an increase in the crystalline nature of the budesonide-loaded hydrogel (19.6%) as compared to the unloaded hydrogel (10.6%), due to the high crystallinity of budesonide. Characteristic peaks of budesonide can be seen in the X-ray diffractogram of the loaded hydrogel, indicating that the hydrogel has been loaded with the drug successfully.

#### 3.2.3. Differential Scanning Calorimetry (DSC)

**Figure 7:**
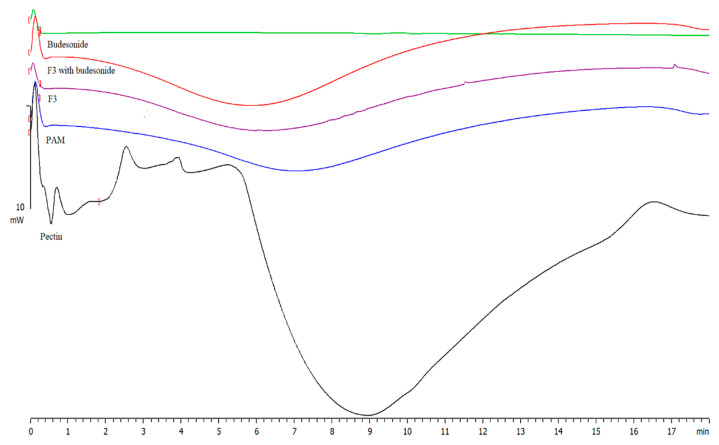
DSC thermogram of pure pectin, polyacrylamide, F3 without the drug, F3 with drug and budesonide.

The DSC thermograms of PC and PC/PAM hydrogel (F3) are shown in Figure 7. In DSC of PC, a small peak at 74 °C was observed. The peak represents the glass transition temperature (Tg) of pure PC. On the other hand, the thermogram of hydrogel showed a peak at temperature 84 °C which corresponds to glass transition temperature (Tg) of the grafted PC. This shows that there is an increase in Tg of the pure PC after blending with PAM. The increase in Tg may be attributed to the presence of intermolecular hydrogen bonding between PC (–OH) and PAM (–C=O), indicating the formation of a grafted polymer. Similarly, the thermogram of PAM showed the occurrence of endothermic transitions around 170 °C. Budesonide did not show any sharp peak as stated in the literature. Therefore, further investigation is required in this context (Figure S2).

#### 3.2.4. Morphological Analysis

**Figure 8:**
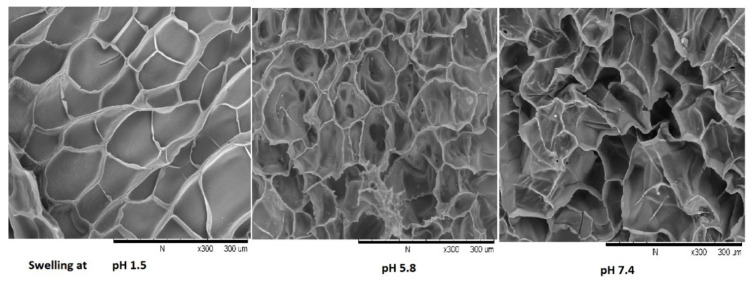
SEM images of the hydrogel at different pH (1.5, 5.8, 7.4) at magnification 300×.

The surface morphology of budesonide hydrogel was investigated by scanning electron microscope at 300× magnification and it was revealed from Figure 8 the surface of hydrogels has interconnected pores at pH 5.8 and pH 7.4, however no pores appeared at acidic pH, which supports the pH-sensitive nature of the hydrogel. Prior studies have reported that interconnected pores and spongy structures allow greater diffusion of solvent molecules during swelling. The presence of a continuous porosity network would also enhance the water sorption capacity of the hydrogel [27]. The porosity plays multiple roles of enhancing the total water sorption capability and the rate of response by reducing the transport resistance. Therefore, the creation of porosity in hydrogels has been considered an important process in many ways [28].

#### 3.2.5. Drug Loading and In-Vitro Drug Release

**Figure 9:**
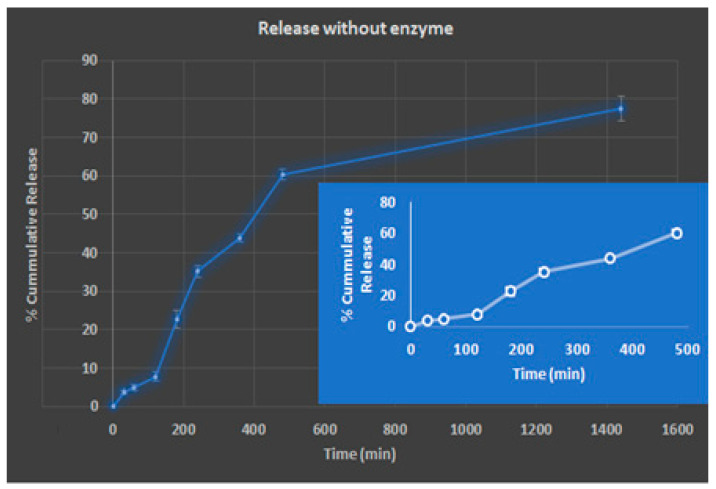
Graph of cumulative percentage drug release over time.

On the basis of gel fraction and swelling, hydrogel-F3 was selected for drug loading (DL) and release. The EE of F3 was 80.4 ± 3.34% respectively, and the DL was 8.89 ± 1.35%, respectively. The in vitro release profile of hydrogel principally depends on percentage swelling of hydrogel in dissolution media, solubility of the drug in media, and interaction of the drug with the polymeric network [29]. The release pattern was estimated in phosphate buffer at hydrochloric acid buffer pH 1.5 for 2 h followed by pH 7.4. The cumulative percentage release in phosphate buffer pH 1.5 during the first 2 h in F3 was 7%, while the cumulative release in pH 7.4 for next 3 h was 36%. The same pattern was observed in total cumulative release (Figure 9), attributable to the swelling behaviour of the hydrogel with varying pH. The drug release pattern was characterised by a biphasic, burst release, resulting from the presence of the drug on the hydrogel surface, followed by sustained release. Initially, the rapid release was observed because the concentration gradient functioned as the driving force for release, followed by a reduced release rate due to the depletion of the drug near the surface. According to Graham and McNeil (1984), hydrogel combines two components, one being a strong polymer network and the other being permeation channels filled with water for the diffusion of water-soluble solute. Thus, the release of the hydrogel mainly governs by the water content of the hydrogel. So, the hydrogel with greater water content is expected to release a higher amount of solute.

The correlation coefficient (R^2^) of F3 was found to be 0.9772, 0.8720, 0.9820, and 0.9390 for zero order, first order, Higuchi, and Korsmeyer-Peppas respectively (Table 3). The data revealed that release pattern of hydrogel was best fitted into zero-order kinetics as confirmed by higher R^2^. This denotes the constant release of drug from hydrogel for stipulated time. Additionally, Higuchi was used to identify the release mechanism and represent diffusion as the dominant release mechanism. As discussed earlier, at basic pH, porous channels formed in hydrogel which leads to the slow diffusion of drug from formulation. Furthermore, the value of the release exponent of Korsmeyer-Peppas (n) in F3 was 0.697, which is between 0.45 and 0.89, which represents the non-Fickian diffusion, indicating that release of the drug via a dominant swelling-controlled diffusion mechanism along with slight surface erosion that occurred concurrently.

## 4. Conclusions

In this study, we have reported the development and optimization of pH-sensitive PC/PAM hydrogel for budesonide by grafting AM on PC with the aid of the initiator potassium persulfate followed by cross-linking with MBA. XRD studies showed the amorphous nature of hydrogel and loading of drug in hydrogel. Moreover, an increase in the crystallinity of the budesonide-loaded hydrogel was observed compared to the unloaded hydrogel, due to the high crystallinity of budesonide. DSC thermograms illustrate the increase in Tg of grafted polymer which is attributed to the presence of an intermolecular hydrogen bond. Additionally, the hydrogel revealed pH-dependent swelling and biphasic release of the drug from the hydrogel. This release pattern showed the burst release of budesonide from the surface followed by sustain release with non-ficken diffusion mechanism. Therefore, PC/PAM hydrogel could be a promising approach for the colon-targeted delivery of budesonide for the treatment of ulcerative colitis. Future scopes of investigation include cytotoxicity and stability studies, along with in-vivo pre-clinical effectiveness.

## Figures and Tables

**Table 1:** Gel fraction of PC/polyacrylamide hydrogel based on different amounts of acrylamide and PC with a constant ratio of MBA/KPS.

Formulation Code for Hydrogel	AM (g)	PC (g)	Ratio of MBA/KPS	Gel Fraction of Hydrogel (%)	SD
1A	1.5	0.5	50/50	58.9	0.63
1B	1.5	1	50/50	49.01	2.17
F3	2.1	0.5	50/50	77.23	1.57
1C	2.1	1	50/50	72.29	0.53
1D	2.8	0.5	50/50	81.08	2.47
1E	2.8	1	50/50	76.09	1.25

**Table 2:** Gel fraction of PC/polyacrylamide hydrogel based on different amounts of initiator and cross linker with constant ratio of AM/PC.

Formulation Code for Hydrogel	Ratio of AM/PC	MBA (mg)	KPS (mg)	Gel Fraction of Hydrogel (%)	SD
F1	2.1/0.5	10	50	60.23	1.03
F2	2.1/0.5	20	50	62.81	0.98
F3	2.1/0.5	50	50	77.23	1.57
F4	2.1/0.5	70	50	78.05	0.86
F5	2.1/0.5	90	50	79.47	1.73
F6	2.1/0.5	50	20	63.4	0.78
F7	2.1/0.5	50	70	79.13	1.18

**Table 3:** Drug release kinetics of drug-loaded hydrogel.

Formulation	Zero-Order (R^2^)	First-Order (R^2^)	Higuchi (R^2^)	Hixon-Crowell (R^2^)	Korsmeyer-Peppas (n)
Drug loaded hydrogel (F3)	0.9772	0.8720	0.9320	0.9390	0.697

## Data Availability

Data is freely available.

## Supplementary Materials

The following are available online, Figure S1: Photos of prepared hydrogels. Figure S2: DSC thermogram of budesonide; budesonide loaded hydrogel; polyacrylamide.

## Sample availability

The data presented in this study are available on request from the corresponding author.
