# Supplementary material for: Budesonide-Loaded Pectin/Polyacrylamide Hydrogel for Sustained Delivery: Fabrication, Characterization and In Vitro Release Kinetics
Source: Molecules. 2021 May 5;26(9):2704. doi: 10.3390/molecules26092704 (PMC8124457; doi:10.3390/molecules26092704)
Supplement: Supplementary file 1 [file molecules-26-02704-s001.zip › molecules-1160936-supplementary.pdf]

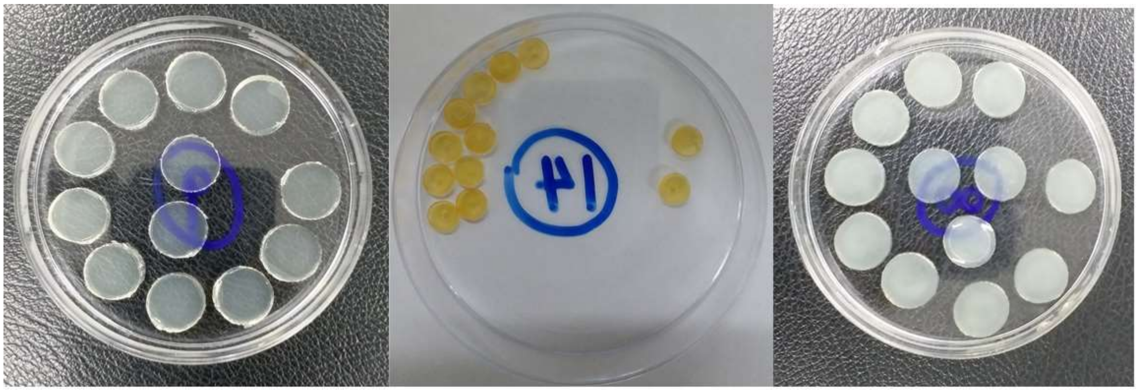

Hydrogel disk (wet) | Dried Hydrogel disk | Drug loaded Hydrogel disk (wet)

Figure S1: Photos of prepared hydrogels.

DSC of budesonide

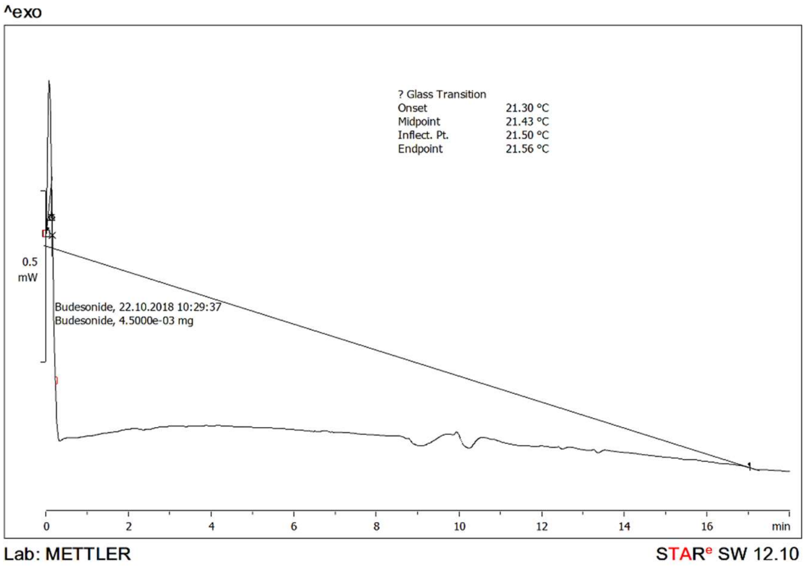

DSC of budesonide loaded hydrogel.

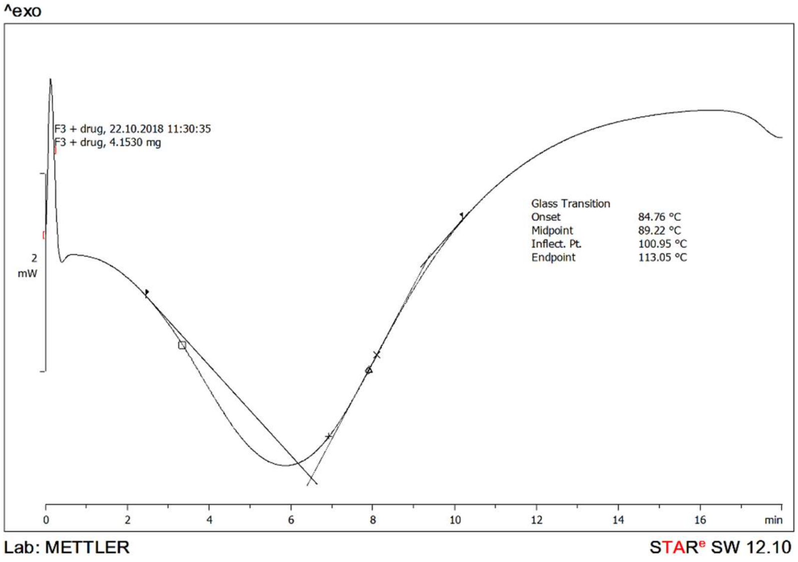

Polyacrylamide

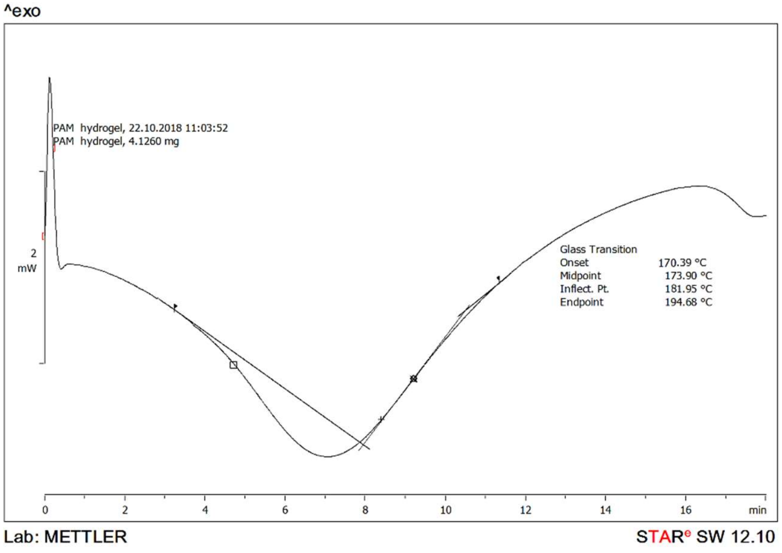

Figure S2: DSC thermogram of budesonide; budesonide loaded hydrogel; polyacrylamide.
